# Honokiol suppresses TNF-α-induced neutrophil adhesion on cerebral endothelial cells by disrupting polyubiquitination and degradation of IκBα

**DOI:** 10.1038/srep26554

**Published:** 2016-05-23

**Authors:** Po-Jen Chen, Yu-Ling Wang, Liang-Mou Kuo, Chwan-Fwu Lin, Chun-Yu Chen, Yung-Fong Tsai, Jiann-Jong Shen, Tsong-Long Hwang

**Affiliations:** 1 Graduate Institute of Natural Products, School of Traditional Medicine, College of Medicine, Chang Gung University, Taoyuan 333, Taiwan; 2 Chinese Herbal Medicine Research Team, Healthy Aging Research Center, Chang Gung University, Taoyuan 333, Taiwan; 3 Graduate Institute of Clinical Medical Sciences, College of Medicine, Chang Gung University, Taoyuan 333, Taiwan; 4 Department of General Surgery, Chang Gung Memorial Hospital, Chiayi 613, Taiwan; 5 Research Center for Industry of Human Ecology and Graduate Institute of Health Industry Technology, Chang Gung University of Science and Technology, Taoyuan 333, Taiwan; 6 Department of Anaesthesiology, Chang Gung Memorial Hospital, Taoyuan 333, Taiwan

## Abstract

Adhesion molecules expressed on cerebral endothelial cells (ECs) mediate leukocyte recruitment and play a significant role in cerebral inflammation. Increased levels of adhesion molecules on the EC surface induce leukocyte infiltration into inflammatory areas and are thus hallmarkers of inflammation. Honokiol, isolated from the Chinese medicinal herb *Magnolia officinalis*, has various pharmacological activities, including anti-inflammatory effects, yet the nature of honokiol targeting molecules remains to be revealed. Here, we investigated the inhibitory effect of honokiol on neutrophil adhesion and vascular cell adhesion molecule-1 (VCAM-1) expression, which underlie its molecular target, and mechanisms for inactivating nuclear factor κ enhancer binding protein (NF-κB) in mouse cerebral ECs. Honokiol inhibited tumour necrosis factor-α (TNF-α)-induced neutrophil adhesion and VCAM-1 gene expression in cerebral ECs. The inflammatory transcription factor NF-κB was downregulated by honokiol. Honokiol significantly blocked TNF-α–induced NF-κB p65 nuclear translocation and degradation of the proteasome-dependent inhibitor of NF-κB α (IκBα). From docking model prediction, honokiol directly targeted the ubiquitin–ubiquitin interface of Lys48-linked polychains. Moreover, honokiol prevented the TNF-α-induced Lys48-linked polyubiquitination, including IκBα-polyubiquitin interaction. Honokiol has protective anti-inflammatory effects on TNF-α-induced neutrophil adhesion and VCAM-1 gene expression in cerebral ECs, at least in part by directly inhibiting ubiquitination-mediated IκBα degradation and then preventing NF-κB nuclear translocation.

Cerebral endothelial cells (ECs) are the major components of the blood–brain barrier (BBB). Cerebral ECs are distinct from peripheral ECs because they have fewer vesicles, higher mitochondrial volume fraction, higher electrical resistance, and unique transport systems^1^,^2^,^3^. They act as a barrier to the central nervous system (CNS) and limit the entry of monocytes, lymphocytes or other leukocytes under physiological conditions.

CNS inflammation is associated with disrupted BBB integrity followed by activated leukocyte transmigration^4^,^5^. Adhesion molecules such as vascular cell adhesion molecule-1 (VCAM-1), intercellular adhesion molecule-1 (ICAM-1) and E-selectin are hallmarkers of inflammation because their increased expression on the EC surface plays a critical role in monocyte and leukocyte infiltration into inflammation areas^6^,^7^. Increased expression of particular adhesion molecules in various inflammatory diseases may suggest opportunities for the development of new therapeutic strategies^8^. VCAM-1 expression is low in normal cerebral ECs but high in ECs during CNS inflammatory disorders such as stroke, multiple sclerosis, and brain tumor metastasis^3^,^9^,^10^,^11^. A therapeutic strategy may be to repress VCAM-1 expression and diminish infiltration of inflammatory cells into the brain.

Tumour necrosis factor-α (TNF-α) is a critical cytokine during the progression of CNS diseases and induces the expression of the adhesion molecules VCAM-1, ICAM-1 and E-selectin contributing to these complex processes^12^,^13^,^14^. The inflammatory transcription factor, nuclear factor κ enhancer binding protein (NF-κB), is responsible for upregulating these pro-inflammatory genes. In the canonical pathway, NF-κB is rapidly activated by various inflammatory cytokines in a wide variety of biological phenomena. With TNF-α stimulation, the inhibitor of NF-κB α (IκBα) is phosphorylated and sequentially conjugated with K48-linked polyubiquitin, which leads to degradation of IκBα by the 26S proteasome. Such degradation allows NF-κB to translocate into nucleus and sequentially stimulate the expression of pro-inflammatory target genes^15^,^16^,^17^. TNF-α-mediated NF-κB activation is tightly controlled by ubiquitination, especially in the IκB pathway^18^,^19^. Hence, interfering with ubiquitination in NF-κB signalling is an interesting strategy for preventing excessive inflammation.

Honokiol (C_18_H_18_O_2_) (Fig. 1A), a natural product isolated from the Chinese medicinal herb *Magnolia officinalis*, has several pharmacological effects because of its anti-angiogenesis, anti-tumor, neuroprotection, and anti-inflammatory properties^20^,^21^,^22^,^23^,^24^,^25^,^26^. Honokiol can easily penetrate the BBB and blood–cerebrospinal fluid barrier^27^, so it may be an effective drug for treating CNS disorders. Honokiol has been extensively studied for cancer prevention and therapy because of its multiple targeted pathologically relevant pathways, including epidermal growth factor receptor, signal transducer and activator of transcription 3, and NF-κB^28^,^29^,^30^. Honokiol can block NF-κB-controlled gene expression such as matrix metalloproteinase-9, TNF-α, interleukin-8, ICAM-1, and monocyte chemotactic protein-1 in various cell types^30^,^31^,^32^. Recently, honokiol was found a natural inhibitor of NF-κB^33^. Honokiol could repress the TNF-α–induced NF-κB activation through an extracellular signal-regulated kinase pathway in rat aortic smooth muscle cells^34^. Although honokiol is a potential natural product for treating CNS disorders, how it terminates NF-κB signalling downstream of the TNF receptor in inflammatory cerebral ECs is unknown.

To elucidate the possible mechanism(s) of the anti-inflammatory effect of honokiol in cerebral ECs, we aimed to investigate how honokiol attenuates a TNF-α–induced inflammatory effect via repressing NF-κB-activated VCAM-1 expression. Honokiol suppressed TNF-α-induced neutrophil adhesion by inhibiting VCAM-1 gene expression in ECs. Furthermore, it inhibited NF-κB p65 nuclear translocation and prohibited it from targeting inflammatory genes by blocking IκBα degradation. Honokiol targeted the ubiquitin–ubiquitin interface of K48-linked polychains, then repressed the TNF-α–induced polyubiquitination of IκBα. Our study reveals another mechanism of honokiol in protecting ECs against inflammation by modulating the polyubiquitination of IκBα to fine-tune NF-κB–activated transcriptional adaption, with potential as an agent for preventing and treating CNS diseases.

## Results

### Honokiol suppressed TNF-α-induced neutrophil adhesion and VCAM-1 expression in ECs

**Figure 1:**
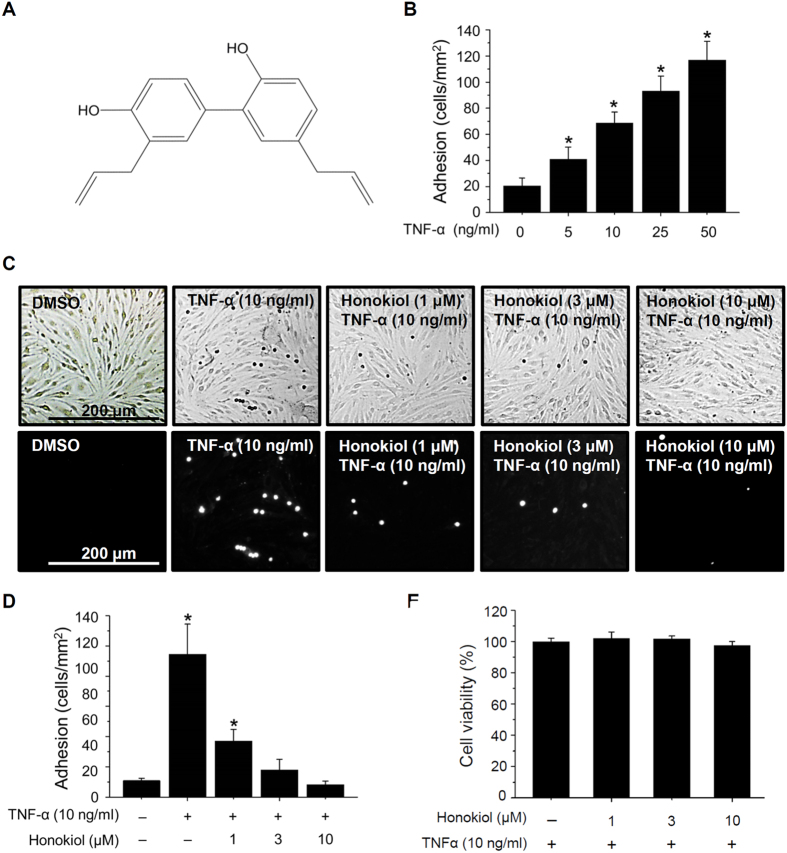
Honokiol repressed tumor necrosis factor α (TNF-α)-induced adhesion of neutrophils to endothelial cells (ECs). (**A**) Chemical structure of honokiol. (**B**) bEnd.3 ECs were incubated with TNF-α at various concentrations (5, 10, 25, and 50 ng/ml) for 3 h. (**C**) Human neutrophils were then incubated with ECs for 30 min. Adherent neutrophils on ECs were detected by microscopy. ECs were incubated with DMSO or honokiol (1, 3, and 10 μM) for 30 min, then stimulated with TNF-α (10 ng/ml) for 3 h. DiI-labelled neutrophils were incubated with ECs for 30 min. Adherent neutrophils on ECs were detected by microscopy. Bars, 200 μm. (**D**) Adherent neutrophils were counted and quantified. (**E**) Cell viability measured by WST-1 reagent in ECs incubated with or without honokiol (1, 3, and 10 μM) for 30 min, then treated with TNF-α (10 ng/ml) for 6 h. Data are mean ± SEM from 5 experiments performed in duplicate. *P < 0.05 vs untreated control cells.

To investigate the anti-inflammatory effects of honokiol on ECs, we first explored whether honokiol inhibited neutrophil adhesion to ECs. Stimulation of ECs with TNF-α (5, 10, 25, and 50 ng/ml) dose-dependently promoted neutrophil adhesion (Fig. 1B). On pre-treating ECs with honokiol (1, 3, and 10 μM) for 30 min, then TNF-α (10 ng/ml), honokiol significantly decreased in the amount of neutrophils on ECs (Fig. 1C,D). Honokiol did not alter EC cell viability with TNF-α (10 ng/ml) treatment for 6 h as demonstrated by viability assay (Fig. 1E), suggesting that honokiol exerted anti-inflammatory effects in ECs without affecting cell viability.

**Figure 2:**
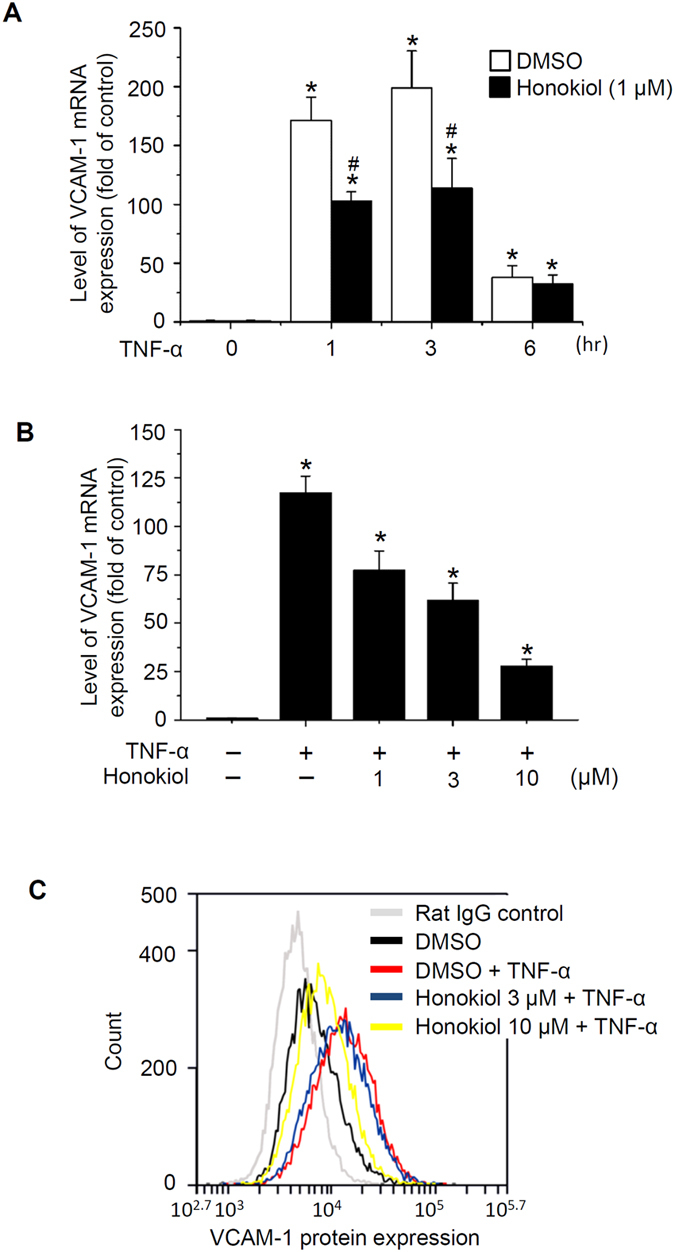
Honokiol suppressed TNF-α-induced adhesion molecule expression. (**A**) RT-qPCR analysis of vascular cell adhesion molecule-1 (VCAM-1) mRNA level in bEnd.3 ECs incubated with DMSO or honokiol (1 μM) for 30 min, then stimulated with TNF-α (10 ng/ml) for 1, 3, and 6 h. Normalization was to β-actin mRNA level. (**B**) RT-qPCR analysis of VCAM-1 mRNA level in ECs incubated with honokiol at 1, 3, and 10 μM for 30 min, then stimulated with TNF-α (10 ng/ml) for 1 h. (**C**) ECs were incubated with DMSO or honokiol (3 or 10 μM) for 30 min, then stimulated with TNF-α (10 ng/ml) for 1 h. ECs were stained with rat IgG or anti-VCAM-1antibodies for 30 min. The unbound antibodies were removed, and then Alexa Fluor 488 anti-rat IgG was added for 30 min. Fluorescein-labelled ECs were analysed for surface VCAM-1 expression by flow cytometry. ECs incubated with rat IgG and Alexa Fluor 488 dye-conjugated antibodies were rat IgG controls. Data are mean  ±  SEM from 3 independent experiments. *P < 0.05 vs untreated control. ^#^*P* < 0.05 vs corresponding control (TNF-α alone).

The cell adhesion molecule VCAM-1 has a critical role in leucocyte adhesion to ECs at the site of CNS inflammation. We treated ECs with TNF-α (10 ng/ml) for various times to investigate VCAM-1 mRNA expression. TNF-α significantly increased VCAM-1 mRNA expression at 1, 3, and 6 h, with peak response at 3 h (Fig. 2A). Honokiol pre-treatment dose-dependently reversed TNF-α–induced VCAM-1 mRNA levels (Fig. 2B). Flow cytometry of EC-surface VCAM-1 protein expression confirmed the decreased VCAM-1 expression in ECs (Fig. 2C). Therefore, honokiol suppressed the TNF-α–induced neutrophil adhesion to ECs, which should depend on downregulation of VCAM-1 expression in ECs.

### Honokiol prevented NF-κB nuclear translocation by inhibiting IκBα degradation

**Figure 3:**
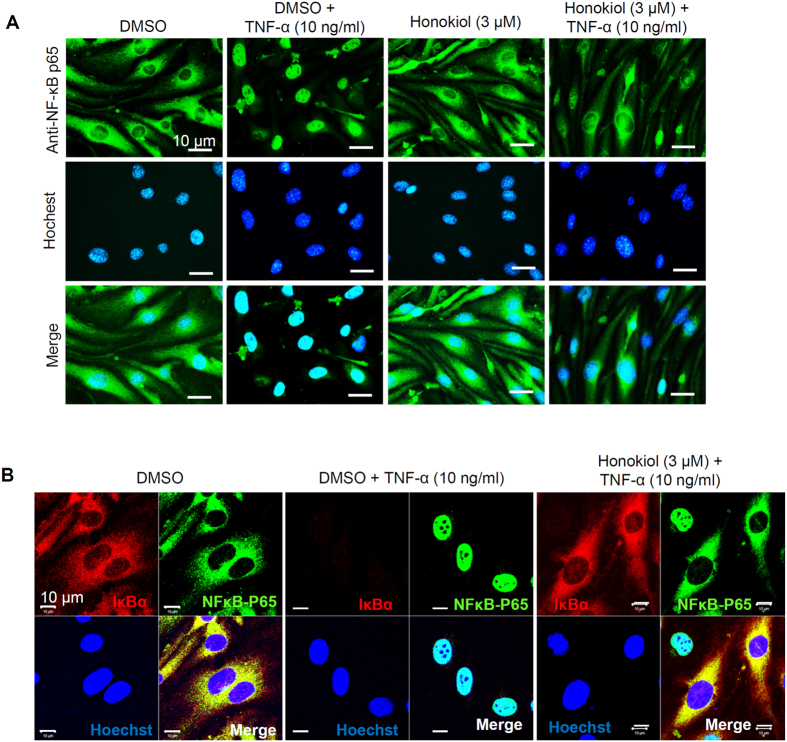
Honokiol blocked the TNF-α-induced NF-κB p65 nuclear translocation and inhibitor of NF-κB α (IκBα) degradation. (**A**) Inverted microscopy of intracellular localization of NF-κB p65 (green) in ECs incubated with DMSO or honokiol (3 μM) for 30 min, then with TNF-α (10 ng/ml) for 15 min. Nuclei were stained with Hoechst 33342 (blue). (**B**) Confocal microscopy of localization and expression of IκBα (Red) and p65 (green) in ECs preincubated with DMSO or honokiol (3 μM) for 30 min, then with TNF-α (10 ng/ml) for 15 min. Bars, 10 μm. Representative images from 3 or 4 experiments are shown.

**Figure 4:**
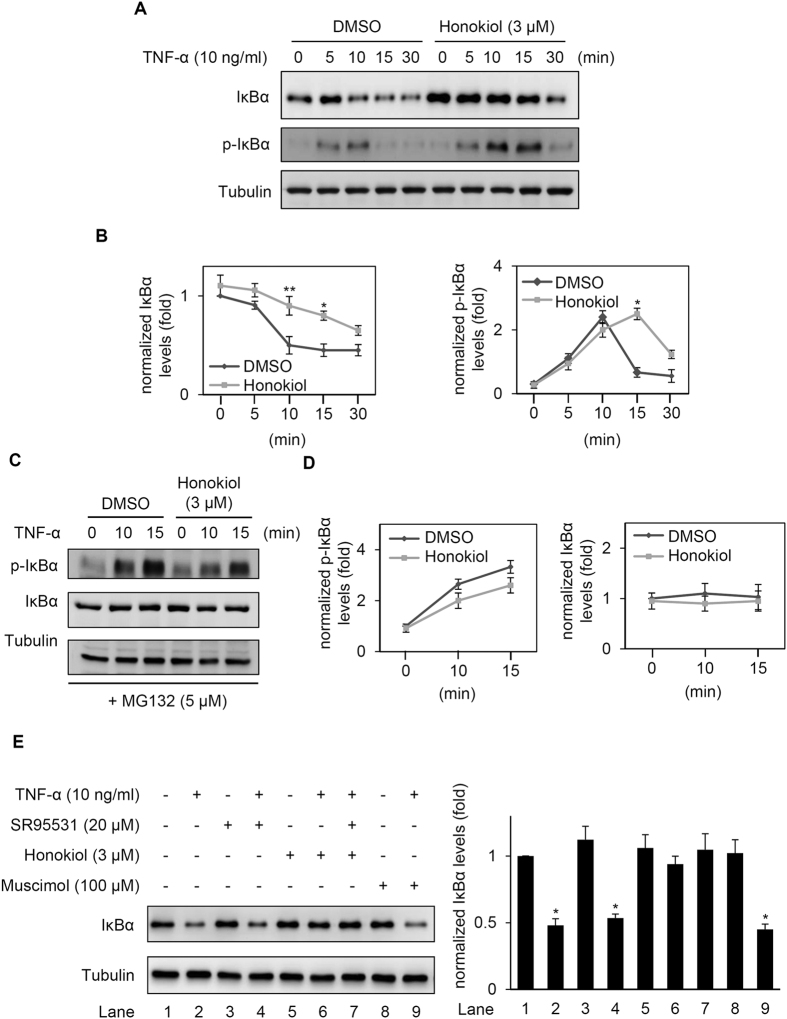
Honokiol prevented the TNF-α-induced IκBα proteosomal degradation. (**A**) Western blot analysis of IκBα, phospho-IκBαand tubulin (loading control) protein expression in ECs pre-treated with DMSO or honokiol (3 μM) for 30 min, then stimulated with TNF-α (10 ng/ml) at different times (5, 10, 15, and 30 min). (**B**) IκBα and phospho-IκBαsignals were quantified, expressed as a relative ratio (normalized with tubulin) and plotted against time. (**C**) Western blot analysis of phosphor-IκBα, IκBα and tubulin protein expression in ECs treated with MG132 (5 μM) for 1 h, then incubated with DMSO or honokiol (3 μM) for 30 min and stimulated with TNF-α (10 ng/ml). (**D**) Phospho-IκBα and IκBα signals were quantified, expressed as a relative ratio (normalized with tubulin) and plotted against time. (**E**) EC cells were exposure to DMSO or the GABA_A_ receptor antagonist, SR95531 (20 μM), for 30 min and sequentially treated with DMSO, honokiol (3 μM), or GABA_A_ agonist, muscimol (100 μM) for 30 min. The ECs were stimulated with TNF-α (10 ng/ml) for 15 min and analyzed using western blot. The IκBα signals were quantified expressed as a relative ratio (normalized with tubulin). Representative images from 3 experiments are shown. Data are mean ± SEM from 3 independent experiments. *P < 0.05; **P < 0.01 versus untreated control cells. All the Western blotting experiments were performed under the following condition. After transferring the blots onto nitrocellulose membranes, we immediately cropped the targeted blots according to referenced indicating markers, and then targeted proteins were immunoblotted with its specific monoclonal antibody.

Because the VCAM-1 gene promoter region contains NF-κB binding sites^35^ and previous studies indicated that honokiol inhibited NF-κB activity^30^,^31^,^33^,^34^, we examined whether honokiol regulated NF-κB signalling in cerebral ECs. NF-κB is a heterodimeric transcription factor including p65 (RelA), RelB, c-Rel, p105/p50, and p100/p52 and is inactivated in the cytoplasm when binding to the inhibitor protein IκB^17^. On treatment with stimuli such as TNF-α, IκB is degraded and causes NF-κB to translocate into the nucleus^17^. Correspondingly, we found that treating ECs with honokiol (3 μM) supressed TNF-α-induced p65 nuclear translocation (Fig. 3A). p65 and IκBα were co-localized in the cytoplasm of control ECs (Fig. 3B). TNF-α (10 ng/ml) treatment for 15 min produced IκBα degradation and p65 nuclear translocation; however, honokiol (3 μM) could prevent the TNF-α-induced IκBα degradation and p65 nuclear translocation (Fig. 3B). Similar results were obtained by western blot analysis: TNF-α–induced IκBα degradation at 10 and 15 min was significantly abolished by honokiol treatment (3 μM) (Fig. 4A,B). Additionally, the TNF-α–induced IκBα phosphorylation was accumulated by honokiol treatment (Fig. 4A,B), suggesting that honokiol may mainly alter on the IκBα stability instead of phosphorylation. To further determine whether the TNF-α–induced IκBα degradation or phosphorylation was affected by honokiol, ECs were treated with MG132, a proteasome inhibitor preventing proteasomal degradation of IκB^36^. MG132 (5 μM) inhibited TNF-α-induced IκBα degradation, thus the TNF-α-activated IκBα phosphorylation was increased. Honokiol had no effect on IκBα expression and slightly suppressed the TNF-α-induced IκBα phosphorylation although there was no statistical significance (Fig. 4C,D). We suggest that honokiol may exert dual effects on TNF-α-controlled IκBα-NFκB pathway through the main polyubiquitin targeting and minor phosphorylation signalling in our model. Additionally, it has been reported that honokiol acts on the GABA_A_ receptor^20^,^37^. To explore whether the effect of honokiol in ECs is dependent on GABA_A_ receptor, the GABA_A_ receptor antagonist, SR95531, or GABA_A_ agonist, muscimol, were tested. As the results shown in Fig. 4E, muscimol did not alter the TNF-α-induced IκBα degradation in ECs. Furthermore, SR95531 did not affect the inhibitory effect of honokiol on TNF-α-induced IκBα degradation in ECs. These results suggested that honokiol-blocked IκBα degradation is not changed by the GABA_A_ receptor antagonist as well as GABA_A_ agonist.

### Honokiol targeted the ubiquitin–ubiquitin interface of K48-linked polychains

**Figure 5:**
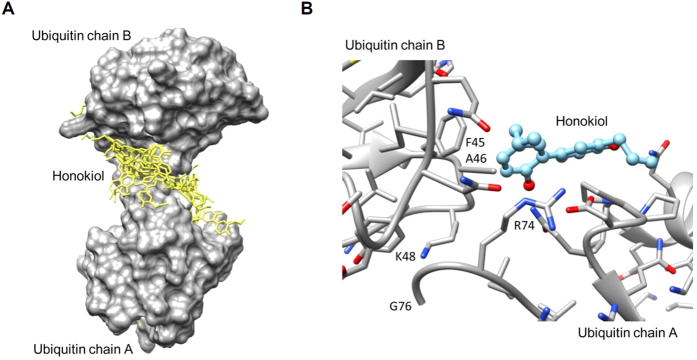
Docking models of honokiol-targeted ubiquitin. (**A**) Surface presentation demonstrates the structure of di-ubiquitin (gray). Honokiol moieties are coloured yellow and rendered in stick representation. (**B**) Close-up of honokiol docking site (best energy mode) in the ubiquitin–ubiquitin interface of Lys48-linked chains. The figures were prepared by using Chimera software. The crystal structure of ubiquitin was obtained from the PDB (accession code 1F9J)^62^. The honokiol 3D structure was obtained from the Zinc database (code 1536, ZINC 12)^63^.

Because IκBα degradation is tightly controlled by ubiquitination^18^, we wondered whether honokiol blocked the ubiquitination of IκBα. Hence, we investigated honokiol docking with K48-linked polyubiquitin. After molecule docking investigations with SwissDock^38^, 31 clusters of binding models were generated. Honokiol moieties were located near the interface of di-ubiquitin in most of the binding models (Fig. 5A). The best energy binding model is in Fig. 5B; the aromatic ring of honokiol can interact with hydrophobic residues F45 and A46 in ubiquitin chain A. R74, located at C-terminal ubiquitin chain B was close to the -OH group of honokiol (Fig. 5B). Therefore, honokiol may bind to the ubiquitin–ubiquitin interface of K48-linked polychains and interfere with the polyubiquitin linkage.

### Honokiol blocked K48-linked polyubiquitination of IκBα in ECs

**Figure 6:**
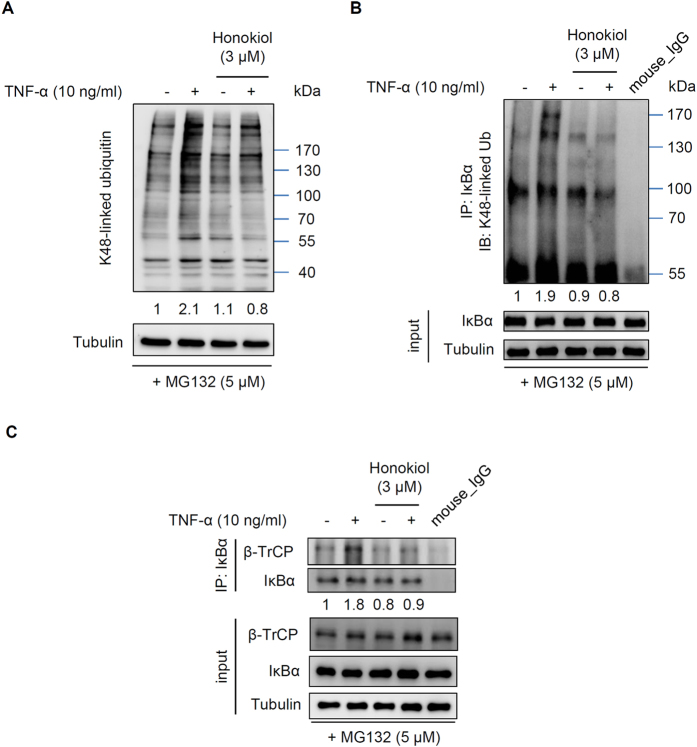
Honokiol blocked TNF-α-induced K48-linked polyubiquitination in ECs. (**A**) Western blot analysis of K48- linked polyubiquitination in the presence and absence of honokiol or TNF-α. The whole ubiquitin signals were quantified expressed as a relative ratio (normalized with tubulin). (**B,C**) Immunoprecipitation and western blot assay of the interaction between K48-linked polyubiquitin (**B**) or β-TrCP (**C**) and IκBα in ECs pre-treated with honokiol (3 μM) for 30 min, then stimulated with TNF-α (10 ng/ml) for 15 min in the presence of MG132 (5 μM). The IκBα-precipitated K48-linked polyubiquitin (100–170 kDa) (**B**) or β-TrCP (**C**) signals were quantified expressed as a relative ratio (normalized with input IκBα). Representative images from 3 experiments are shown.

TNF-α-mediated NF-κB signal transduction is regulated by ubiquitination of various proteins via K48-, K63- or M1 (linear)-linked polychains^39^,^40^. To further confirm our docking models in Fig. 5, we determined whether honokiol inhibited IκBα degradation by directly blocking K48-linked polyubiquitination of IκBα in ECs. By western blot analysis, pretreating ECs with honokiol (3 μM) reduced the TNF-α–induced K48- but not K63- and linear-linked ubiquitin conjugation (Fig. 6A and Supplemental Fig. S1). TNF-α induced the K48-linked polyubiquitin formation with a 2-fold increase that was repressed by honokiol (Fig. 6A). As well, honokiol markedly blocked the 2-fold increase in conjugation of K48-linked polyubiquitin to IκBα induced by TNF-α on immunoprecipitation assay (Fig. 6B). Additionally, the TNF-α signalling triggers the polyubiquitination of IκBα through the ubiquitin E3 ligase complex SCF-β-TrCP^41^. Honokiol also repressed the 2-fold increase in conjugation of β-TrCP to IκBα induced by TNF-α (Fig. 6C). Together, we suggest that honokiol would block TNF-α-induced IκBα degradation by directly disrupting K48-linked polyubiquitination in ECs.

## Discussion

CNS inflammation is associated with disrupted cerebral EC homeostasis and BBB integrity, followed by activated leukocyte transmigration. The critical pro-inflammatory factor, TNF-α, upregulates adhesion molecules in ECs and facilitates leukocyte extravasation into the CNS^12^,^13^. Thus, an attractive therapeutic approach for treating CNS disorders is to limit leukocyte extravasation into the CNS by targeting endothelial adhesion molecules. NF-κB is considered a critical inflammatory transcription factor upregulating adhesion molecules^13^,^14^. In the present study, honokiol diminished TNF-α–induced adhesion between neutrophils and ECs by blocking VCAM-1 mRNA expression and NF-κB activation in cerebral ECs. This inhibition resulted from directly targeting K48-linked polyubiquitination to prevent IκBα degradation. Such a unique mechanism explains why honokiol may be a potential natural compound for treating CNS inflammation.

The pro-inflammatory cytokine, TNF-α, can induce neutrophil infiltration into inflammatory regions^12^,^13^. Here, we show that TNF-α triggered neutrophil adhesion on ECs via NF-κB activation and VCAM-1 upregulation in ECs (Figs 1, 2, 3). However, TNF-α is also an important cytokine activating neutrophils during inflammation and triggering intracellular signal transduction, including the NF-κB pathway, which plays a leading role in the function of neutrophils^42^,^43^,^44^. In Fig. 1D, although the ECs were pre-treated with TNF-α and honokiol before neutrophils adhesion, the effects of TNF-α and honokiol on neutrophils still should be noted. VCAM-1 in ECs of BBB mediates leukocyte transmigration during inflammation by interacting with α_4_β_1_ integrin on leukocytes^45^,^46^. Also, co-stimulating integrin with cytokines induces NF-κB activation and IκB degradation in neutrophils^47^, so TNF-α-induced cell adhesion may be regulated in ECs and neutrophils. Honokiol has an anti-inflammatory effect in neutrophils by inhibiting reactive oxygen species production and neutrophil infiltration *in vivo*^48^,^49^,^50^. Whether honokiol blocks neutrophil-associated diseases by repressing inflammation-induced NF-κB activation could be addressed.

How TNF-α activates NF-κB pathways in cells has been established^14^,^18^,^19^. In general, the binding of TNF to its receptor (TNFR1) leads to recruitment of several ubiquitin E3 ligases to catalyze K63-linked polyubiquitination of receptor-interacting protein (RIP1), which clusters the transforming growth factor-β-activated kinase 1 (TAK1) and IκB kinase (IKK) complex. TAK1 phosphorylation of IKKα/β triggers IκBα phosphorylation and sequential K48-linked polyubiquitination and degradation of IκBα to activate NF-κB. Here, we showed that honokiol may inhibit IκBα degradation by directly binding to and disrupting K48-linked polyubiquitin, thereby suppressing substrate digestion by the proteasome (Figs 4, 5, 6). However, honokiol has been found to suppress NF-κB activation by regulating IKK-IκBα phosphorylation signalling^30^,^31^. RIP1 is conjugated with K63-linked polyubiquitin following TNF stimulation, then becomes deubiquitinated by A20 through the N-terminal OTU domain of A20. RIP1 is then convertibly conjugated with K48-linked polyubiquitin by the C-terminal zinc finger domain of A20, thereby leading to the proteasomal degradation of RIP1 and inactivation of IKK^14^,^51^. Thus, IKK-IκBα phosphorylation signalling should be affected by upstream ubiquitination. If honokiol also interferes with K48-linked polyubiquitin of RIP1, it should prevent RIP1 degradation and cause IKK activation and IκBα degradation, which would conflict with honokiol-repressed IκBα degradation (Fig. 4). Thus, we focused on the ubiquitination rather than phosphorylation of IκBα in this study, and the detailed association needs further elucidation. In addition, the IKK complex is also activated by K63/M1-linked hybrid ubiquitin chains^39^,^40^. However, cells that cannot generate K63-linked polyubiquitin can still activate IKK and NF-κB with TNF stimulation^52^, which suggests that K63 polyubiquitination-mediated phosphorylation and activation of IKK-IκBα pathway may be not essential for NF-κB activity. As we show in Figs 5 and 6, TNF-α mainly induced K48- but not K63- or linear (M1)-linked polyubiquitin, and honokiol also interfered with K48- but not K63- or linear (M1)-linked polyubiquitin, so honokiol-targeted K48 polyubiquitination may be more dominant in TNF-induced CNS inflammation.

Furthermore, honokiol has been reported to act on the GABA_A_ receptor^20^,^37^. In Fig. 4E, we found that muscimol did not alter the TNF-α-induced IκBα degradation and the GABA_A_ receptor antagonist, SR95531, also did not affect the inhibitory effect of honokiol on TNF-α-induced IκBα degradation in ECs, suggesting that honokiol-blocked IκBα degradation is not changed by the GABA_A_ receptor antagonist as well as GABA_A_ agonist, although muscimol and SR95531 does not address the benzodiazepine binding site of the GABA_A_ receptor. In addition, we found that TNF-α-induced NF-κB p65 nuclear translocation was not completely blocked by honokiol in Fig. 3, however, honokiol significantly inhibited the TNF-α-triggered IκBα degradation at this time point (Figs 3B and 4A). It has been reported that the p65 nuclear translocation was controlled by phosphorylation at Ser536 of itself which was independent on IκBα^53^, suggesting that honokiol may not totally control the TNF-α-regulated NF-κB activity because of IκBα-independent regulation. On the other hand, the TNF-α still caused the IκBα degradation in the presence of honokiol after longer 30 min stimulation in Fig. 4A, suggesting that the effect of honokiol on TNF-α-mediated NF-κB activation may be a delayed response rather than a merely suppressed response, although honokiol would repress the VCAM-1 expression or neutrophil adhesion after 1–3 h TNF-α stimulation (Figs 1 and 2).

Other small compounds, ubistatins, could prevent the polyubiquitination and degradation of Sic1; NMR structures demonstrated that ubistatins blocked proteasome-dependent degradation by binding the ubiquitin–ubiquitin interface of K48-linked polychains^54^. Similarly, our docking models revealed that honokiol may target the ubiquitin–ubiquitin interface of K48-linked chains (Fig. 5A). The hydrophobic interaction between the aromatic ring of honokiol and F45 or A46 may promote a conformational change of the ubiquitin loop. Simultaneously, R74 located at ubiquitin chain B may connect to the hydroxyl group of honokiol by a hydrogen bond, for an orientation change at the C-terminal ubiquitin chain A (Fig. 5B). Honokiol may induce a conformational change causing K48 not to bind to G76 and disrupt K48-linked polyubiquitin linkage, thereby directly interfering with K48-linked ubiquitination in ECs. Furthermore, co-crystallizing the complex of honokiol with various linked ubiquitins including K48- and K63-linked and linear chain ubiquitins may help to understand the mechanistic basis of the biological action of honokiol on ubiquitination. Moreover, honokiol may block the TNF-α signalling-triggered the polyubiquitination of IκBα through the ubiquitin E3 ligase complex SCF-β-TrCP^41^ (Fig. 6B,C). The detail mechanism of honokiol-blocked polyubiquitin via affecting the interaction between IκBα and ubiquitin ligase as well as deubiquitin enzyme may need further investigation.

Currently, there is great interest in developing new drugs from medicinal plants; however, which protein or protein group can be targeted by drugs is difficult to determine. In this study, we demonstrate that the small natural product honokiol is a unique example of disrupting the ubiquitin–ubiquitin interface of K48-linked polychains. Thus, honokiol could prevent IκBα degradation after TNF-α treatment, then the constitutively expressed IκBα prohibit NF-κB from translocating to the nucleus and activating inflammatory genes such as VCAM-1 in ECs. Our study supports an additional novel pharmacological mechanism of honokiol in anti-inflammation and understanding the molecular mechanism of honokiol in cells to enhance its efficiency. Toxicological studies of honokiol treatment have demonstrated no pathologic changes in organs, including liver, lung, kidney, spleen, brain, heart, pancreas, intestines, or bone marrow, after systemic or oral administration^55^. Honokiol also shows high ability to penetrate the BBB^27^. Honokiol is a potential natural product for preventing and treating CNS inflammatory diseases.

In summary, we reveal how honokiol prevents TNF-α–induced neutrophil adhesion on cerebral ECs by disrupting the polyubiquitination and degradation of IκBα to block NF-κB**-**controlled VCAM-1 expression.

## Methods

### Extraction and isolation of honokiol

The dried bark of *Magnolia officinalis* cortex (3 kg) was repeatedly extracted with MeOH (20 L × 3) at 70 °C for 4 h. The methanol extracts were evaporated and partitioned between CH_2_Cl_2_ and H_2_O. The CH_2_Cl_2_ layer was subjected to column chromatography over silica gel and gradient elution with hexane-EtOAc and EtOAc-MeOH mixtures used as solvent systems. The fraction eluted with hexane-EtOAc (10:1–4:1) was repeatedly chromatographed with silica gel and Sephadex LH-20 to obtain honokiol (6.8 g)^56^. The structure of honokiol was determined by ^1^H NMR (CDCl_3_, 500 MHz) spectrum analysis (Supplemental Fig. S1) and compared with reference data^57^. When honokiol was dissolved in dimethyl sulfoxide (DMSO), the final concentration of DMSO in the cell experiments is 0.1% and did not affect the parameters measured.

### Reagents

Rat monoclonal antibodies (mAbs) against VCAM-1 (NBP1-26587) were from Novus biologicals (Littleton, Colorado). Rabbit mAbs against NF-κB p65 (#8242), K48-linkage specific polyubiquitin (#8081), β-TrCP (#4394) and α/β-Tubulin (#2148) as well as mouse mAbs against IκBα (#4814) and phospho-IκBα (Ser32/36) (#9246) were from Cell Signaling Technology (Beverly, MA). Rabbit mAbs against K63-linkage specific polyubiquitin (05–1308) and linear ubiquitin (MABS199) were from Millipore (Temecula, CA). Recombinant mouse TNF-α, rat IgG and mouse IgG_1_ was from R&D systems (Minneapolis, MN). Muscimol was from Tocris Bioscience (Ellisville, MO). All other reagent-grade chemicals were from Sigma (St Louis, MO), including MG132 and SR95531.

### Cell culture

Mouse brain microvascular ECs (bEnd.3) were purchased from the Bioresource Collection and Research Centre (Hsinchu, Taiwan). Cultures were maintained in a humidified atmosphere (37 °C, 5% CO_2_) with Dulbecco’s modified Eagle’s medium (DMEM, Gibco, Grand Island, NY) supplemented with 10% fetal bovine serum (FBS; Biological Industries, Israel) and 100 U/ml penicillin/streptomycin (Gibco). ECs were grown to confluence and passaged every 3 days at 1 × 10^5 ^cells/ml.

### Neutrophil isolation

Human neutrophils were isolated from heparinised peripheral venous blood by using a standard method of dextran sedimentation and Ficoll–Hypaque centrifugation^58^,^59^,^60^,^61^. After hypotonic lysis of contaminating erythrocytes, isolated neutrophils were suspended in RPMI 1640 containing 0.1% FBS. Blood was collected from healthy volunteers (ages 20–30 years). This study protocol was investigated and approved by the Institutional Review Board at Chang Gung Memorial Hospital, and written informed consent was obtained from every volunteer. The methods were carried out in accordance with the approved guidelines.

### Neutrophil adhesion experiment

Neutrophils were labelled with 1,1′-dioctadecyl-3,3, 3′,3′-tetramethylindocarbocyanine (DiI; Molecular Probes, Eugene, OR) for 20 min in RPMI 1640 containing 0.1% FBS. ECs were treated with DMSO or honokiol (1, 3, and 10 μM) for 30 min before TNF-α (5, 10, 25, and 50 ng/ml) for 3 h. The labelled neutrophils (1 × 10^5 ^cells/mL) were then incubated with ECs for 30 min. Non-adherent neutrophils were removed by gentle washing with RPMI. Adherent neutrophils on ECs were counted in 6 randomly selected areas (0.572 mm^2^) under a motorised inverted microscope (IX81, Olympus, Japan) with 10X objective.

### Cell viability assay

Cell viability was measured by WST-1 assay (Roche, Mannheim, Germany). ECs were seeded into 96 wells for 24 h at 5 × 10^4 ^cells/well in 100 μl culture medium and treated with or without honokiol (1, 3, and 10 μM) for 30 min before TNF-α (10 ng/ml) for 6 h, then WST-1 (10 μl) was added and absorbance was measured at 450 nm. The reference wavelength was 600 nm.

### RNA isolation and quantitative real-time PCR

Total RNA was extracted from the ECs by using TRIzol reagent (Invitrogen, Carlsbad, CA) and used as a template for cDNA synthesis. cDNA, obtained with High Capacity cDNA Reverse Transcription Kits (Applied Biosystems, Foster City, CA), was amplified by PCR on a CFX Connect (Bio-Rad) with Power SYBR Green PCR Master Mix (Applied Biosystems) and primers for VCAM-1 (100 nM, sense: 5′-ACGTCAGAACAACCGAATCC-3′; antisense: 5′-GTGGTGCTGTGACAATGACC-3′) and β-actin (sense: 5′-CTGGGTCATCTTTTCACGGT-3′; antisense: 5′-TGTTACCAACTGGGACGACA-3′) used as an internal control. The real-time PCR was conducted at 95 °C for 10 min followed by 40 cycles of denaturation at 95 °C for 10 sec, annealing/extension at 60 °C for 1 min. PCR conditions were optimised to achieve a single peak by melting curve analysis on CFX Connect (Bio-Rad). Raw data collected by use of CFX Manager were analysed and quantified by the relative standard curve method.

### Immunofluorescence with flow cytometry

ECs (5 × 10^6^/mL) were detached with Versene buffer containing EDTA and suspended in phosphate buffered saline (PBS) containing rat IgG control or rat monoclonal antibodies against VCAM-1 (10 μg/mL) at 4 °C for 30 min. The unbound antibodies were removed, then Alexa Fluor 488 anti-rat IgG (Invitrogen) was added for 30 min at 4 °C in PBS containing 10% FBS. Fluorescein-labelled ECs were analysed for surface VCAM-1 expression by flow cytometry (BD Accuri C6, BD Biosciences, San Jose, CA). ECs incubated with rat IgG and Alexa Fluor 488 dye-conjugated antibodies were rat IgG controls.

### Immunofluorescence and microscopy

ECs were grown on coverslips and fixed in 4% paraformaldehyde at room temperature for 15 min. Cells were blocked and permeabilised for 30 min with a solution of PBS containing 0.2% Triton X-100 and 1% bovine serum albumin. After incubation with primary antibodies (p65; 1:200 and IκBα; 1:100, Cell Sgnaling) for 1 h, ECs were washed and incubated with secondary antibodies (Alexa Fluor 488 or 594; 1:500, Molecular Probes) for 30 min. After the addition of Hoechst 33342 (1 μg/ml, Invitrogen) for another 5 min, cells were washed and mounted with prolong antifade reagent (invitrogen) on coverslips. Fluorescence was captured under a motorised inverted microscope (IX81, Olympus) and confocal microscope (LSM510, Carl Zeiss). We used a 63×/NA 1.4 or 100×/NA 1.4 oil objectives (Carl Zeiss). Images were obtained by using LSM 510 META (Carl Zeiss).

### Immunoprecipitation and immunoblot analysis

Cell lysates were prepared in lysis buffer (50 mM Tris-HCl, 150 mM NaCl, 0.5% Nonidet P-40, 5 mM EDTA, 50 mM NaF containing protease and phosphatase inhibitors (Roche) and rotated at 4 °C for 30 min. Protein concentration were determined by using a BCA protein assay kit (Pierce, Rockford, IL). Immunoprecipitation involved 200 μg cell lysates and 0.5 μg mouse IgG1 or anti-IκBα antibodies with overnight incubation at 4 °C, then 100 μl protein G plus/protein A agarose (Calbiochem) added for 12 h with rotation at 4 °C. Beads were washed 4 times with lysis buffer. Bound proteins were eluted by boiling for 10 min in sample buffer, and eluted samples were loaded onto an 8% SDS-PAGE gel. The corresponding lysate (30 μg protein) was loaded onto an SDS-PAGE gel (10% running, 4% stacking) and transferred to PVDF membranes (Millipore), which were blocked with 5% milk in TBS, 0.1% Tween 20 (TBST), then incubated with the designated antibodies and horseradish peroxidase-conjugated secondary antibodies (Cell Signaling). Peroxidase activity was evaluated by using an enhanced chemiluminescence kit (Millipore). The reactive band intensities were analysed by using UVP Biospectrum (UVP LLC, Upland, CA).

### Molecular docking

Small molecules were docked and analyzed on target proteins by using SwissDock, a small molecule-protein docking web service established on EADock DSS^38^. The binding of honokiol and ubiquitin with the most favourable energy was estimated with FACTS and clustered. The crystal structure of ubiquitin was obtained from the PDB (accession code 1F9J)^62^. The 3D structure of honokiol was obtained from the Zinc database (code 1536, ZINC 12)^63^. The predicted binding modes were presented by using UCSF Chimera (a visualisation system for exploratory research and analysis)^64^.

### Statistical analysis

Data are expressed as mean ± SEM. Statistical analysis involved Student’s *t* test for two groups and one-way ANOVA followed by Scheffe’s test for multiple comparisons. *P* < 0.05 was considered statistically significant.

## Additional Information

**How to cite this article**: Chen, P.-J. *et al*. Honokiol suppresses TNF-α-induced neutrophil adhesion on cerebral endothelial cells by disrupting polyubiquitination and degradation of IκBα. *Sci. Rep.* **6**, 26554; doi: 10.1038/srep26554 (2016).

## Supplementary Material

Supplementary Information
