# Supplementary material for: Honokiol suppresses TNF-α-induced neutrophil adhesion on cerebral endothelial cells by disrupting polyubiquitination and degradation of IκBα
Source: Sci Rep. 2016 May 23;6:26554. doi: 10.1038/srep26554 (PMC4876378; doi:10.1038/srep26554)

Honokiol suppresses TNF-α-induced neutrophil adhesion on cerebral endothelial cells by disrupting polyubiquitination and degradation of IκBα

Po-Jen Chen[1,2,+], Yu-Ling Wang[1,2,+], Liang-Mou Kuo[3,4,+], Chwan-Fwu Lin[5], Chun-Yu Chen[3,6], Yung-Fong Tsai[3,6], Jiann-Jong Shen[1,2], Tsong-Long Hwang[1,2,5,6*]

[1]Graduate Institute of Natural Products, School of Traditional Medicine, College of Medicine, Chang Gung University, Taoyuan 333, Taiwan

[2]Chinese Herbal Medicine Research Team, Healthy Aging Research Centre, Chang Gung University, Taoyuan 333, Taiwan

[3]Graduate Institute of Clinical Medical Sciences, College of Medicine, Chang Gung University, Taoyuan 333, Taiwan

[4]Department of General Surgery, Chang Gung Memorial Hospital, Chiayi 613, Taiwan

[5]Research Center for Industry of Human Ecology and Graduate Institute of Health Industry Technology, Chang Gung University of Science and Technology, Taoyuan 333, Taiwan.

[6]Department of Anaesthesiology, Chang Gung Memorial Hospital, Taoyuan 333, Taiwan

[+] These authors contributed equally to this work.

***Address correspondence to:** Dr. Tsong-Long Hwang, Graduate Institute of Natural Products, College of Medicine, Chang Gung University, 259 Wen-Hwa 1st Road, Kweishan, Taoyuan 333, Taiwan. Fax: +886 3 2118506; E-mail: htl@mail.cgu.edu.tw

Supplemental Fig. S1 The structure of honokiol was determined by ^{1}H NMR ($CDCl_3$, 500 MHz) spectrum analysis.

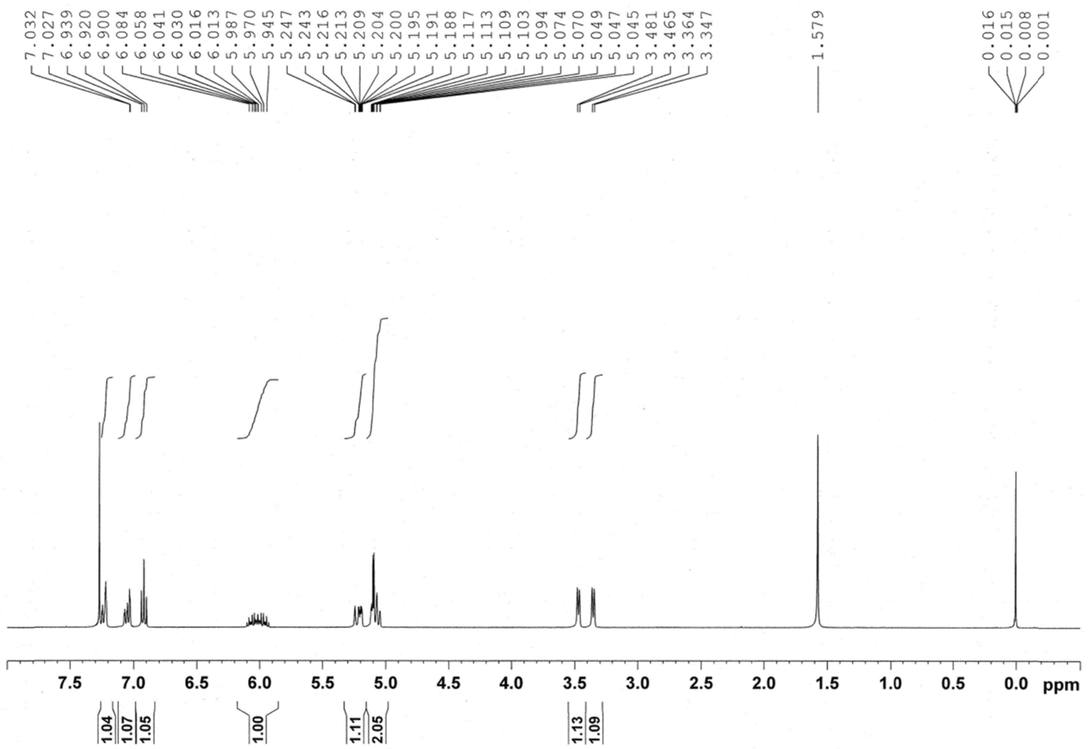

Supplemental Fig. S2 The effect of honokiol on K63- and linear-linked polyubiquitination in ECs. Western blot analysis of K63- and linear-linked polyubiquitination in the presence and absence of honokiol or TNF-α. The whole ubiquitin signals were quantified expressed as a relative ratio (normalized with tubulin).

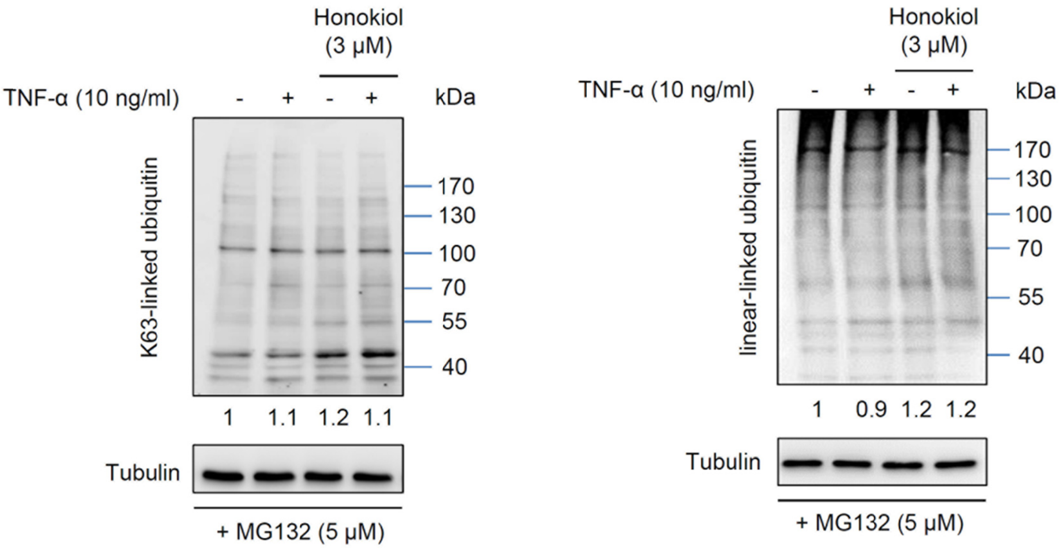

Supplement: Supplementary Information [file srep26554-s1.pdf]
